# A Pilot Study Assessing Left Ventricle Diastolic Function in the Parasternal Long-axis View

**DOI:** 10.5811/westjem.21272

**Published:** 2024-11-21

**Authors:** Mümin Murat Yazici, Nurullah Parça, Enes Hamdioğlu, Meryem Kaçan, Özcan Yavas¸i, Özlem Bilir

**Affiliations:** Recep Tayyip Erdoğan University Training and Research Hospital, Department of Emergency Medicine, Rize, Türkiye

## Abstract

**Introduction:**

Spectral Doppler echocardiography is used to evaluate diastolic dysfunction of the heart. However, it is difficult to assess diastolic function with this modality in emergency department (ED) settings. Based on the hypothesis that E-point septal separation (EPSS) measured by M-mode in the parasternal long-axis (PSLA) view may facilitate the assessment of diastolic function in emergency patient care, we aimed to investigate whether EPSS measured by M-mode in the PSLA view correlates with spectral Doppler assessment in patients with grade 1 diastolic dysfunction.

**Methods:**

We performed this prospective, observational, single-center study was performed in the ED of a tertiary training and research hospital. All patients who presented to the emergency critical care unit with symptoms of heart failure were evaluated by the cardiology department, had grade 1 diastolic dysfunction confirmed by the cardiology department, and did not meet any of the study’s exclusion criteria. The study population of 40 (included rate 14%) was formed after the exclusion criteria were applied to 285 patients who met these conditions. Patients included in the study underwent spectral Doppler measurements in the apical four-chamber (A4C) view followed by M-mode measurements in the PSLA view. We then compared the measurements.

**Results:**

The correlation between the early diastolic velocity of the mitral inflow to the late diastolic velocity (E/A) ratio in spectral Doppler measurements and the EPSS/ A-point septal separation (APSS) ratio in M-mode was strong (correlation coefficient 0.677, *P* = 0.001). Similarly, the correlation between E in spectral Doppler measurements and the EPSS/APSS ratio in M-mode measurements was also moderately strong (correlation coefficient 0.557, *P* = 0.001).

**Conclusion:**

A significant correlation exists between the M-mode EPSS/APSS ratio measurement in the PSLA view and the spectral Doppler E/A ratio measurement in the A4C window to evaluate grade 1 diastolic dysfunction. This association suggests that M-mode measurements in the PSLA may be used in diastolic dysfunction.

Population Health Research Capsule What do we already know about this issue?

*The use of Doppler measurements in the evaluation of diastolic function in the ED is difficult. Therefore, it is necessary to assess diastolic function by a practical method.*

What was the research question?

*Do measurements made by E-point septal separation (EPSS) assessment with M-mode in parasternal long-axis (PSLA) view correlate with those made by spectral Doppler in patients with grade 1 diastolic dysfunction?*

What was the major finding of the study?

*The correlation between the early diastolic velocity of mitral inflow to late diastolic velocity (E/A) ratio in spectral Doppler and the EPSS/ A-point septal separation (APSS) ratio in M-mode was strong (correlation coefficient 0.677, P = 0.001. This suggests that M-mode measurements in the PSLA may be used in diastolic dysfunction.*

How does this improve population health?

*The ability to use M-mode measurements in PSLA for diastolic dysfunction enables rapid diagnosis and prompt treatment of patients in the ED.*

## INTRODUCTION

Point-of-care ultrasound (POCUS) is of vital importance for assessing heart functions in the emergency department (ED).^1^ ^–^ ^3^ Bedside echocardiography, a component of POCUS, is a non-invasive imaging technique used to obtain real-time images of the heart. In this way, systolic and diastolic cardiac function can be evaluated in the ED.^4^ ^,^ ^5^ Although there are many echocardiographic methods for assessing systolic function, the E-point septal separation (EPSS) method, which measures the distance between the ventricular septal wall and the anterior leaflet of the mitral valve (MV) in the parasternal long axis (PSLA) view, is used in the ED to assess the systolic function of the heart. The EPSS method is reliable and simple and does not require specialized equipment or complex calculations. It is particularly useful in emergency patient care.^6^ ^–^ ^8^

The bedside use of the American Society of Echocardiography (ASE) and European Association of Cardiovascular Imaging (EACI) guidelines for the assessment of diastolic function using echocardiography is difficult in in the ED setting is difficult and challenging in many respects.^9^ In spectral Doppler echocardiography various parameters, such as the ratio of the early diastolic velocity of the mitral inflow to the late diastolic velocity (E/A), the E deceleration time, and the ratio of the early diastolic velocity of the mitral inflow to the early diastolic velocity of the mitral annulus (E/e′), are evaluated in the apical four-chamber view (A4C) to assess the diastolic function of the heart.^10^ ^,^ ^11^ Evaluating diastolic function using this spectral Doppler method is simply not practical in emergency patient care.

Various studies have examined the evaluation of diastolic dysfunction by emergency physicians using spectral Doppler echocardiography.^12^ ^,^ ^13^ Some researchers have observed that the E/A pattern evaluated with pulsed wave (PW) Doppler in the A4C view is similar to the motion pattern of the MV anterior leaflet seen when measuring EPSS with M-mode in the PSLA view.^14^ ^,^ ^15^ Based on the hypothesis that this similarity may facilitate the assessment of diastolic function in emergency patient care, we aimed to investigate whether EPSS measured with M-mode in PSLA view correlates with spectral Doppler assessment in patients with grade 1 diastolic dysfunction.

## METHODS

### Study Design and Setting

This prospective, observational, single-center study was performed in the emergency department of a tertiary training and research hospital in Türkiye between December 1, 2023–March 31, 2024. Local ethical committee approval was granted prior to commencement (decision no. 2023/259). All patients we planned to include in the study were told how and for what purpose bedside ultrasonography would be performed, and written informed consent was obtained from all patients who consented to be included in the study.

### Patient Selection and Data Collection

**Figure 1.**
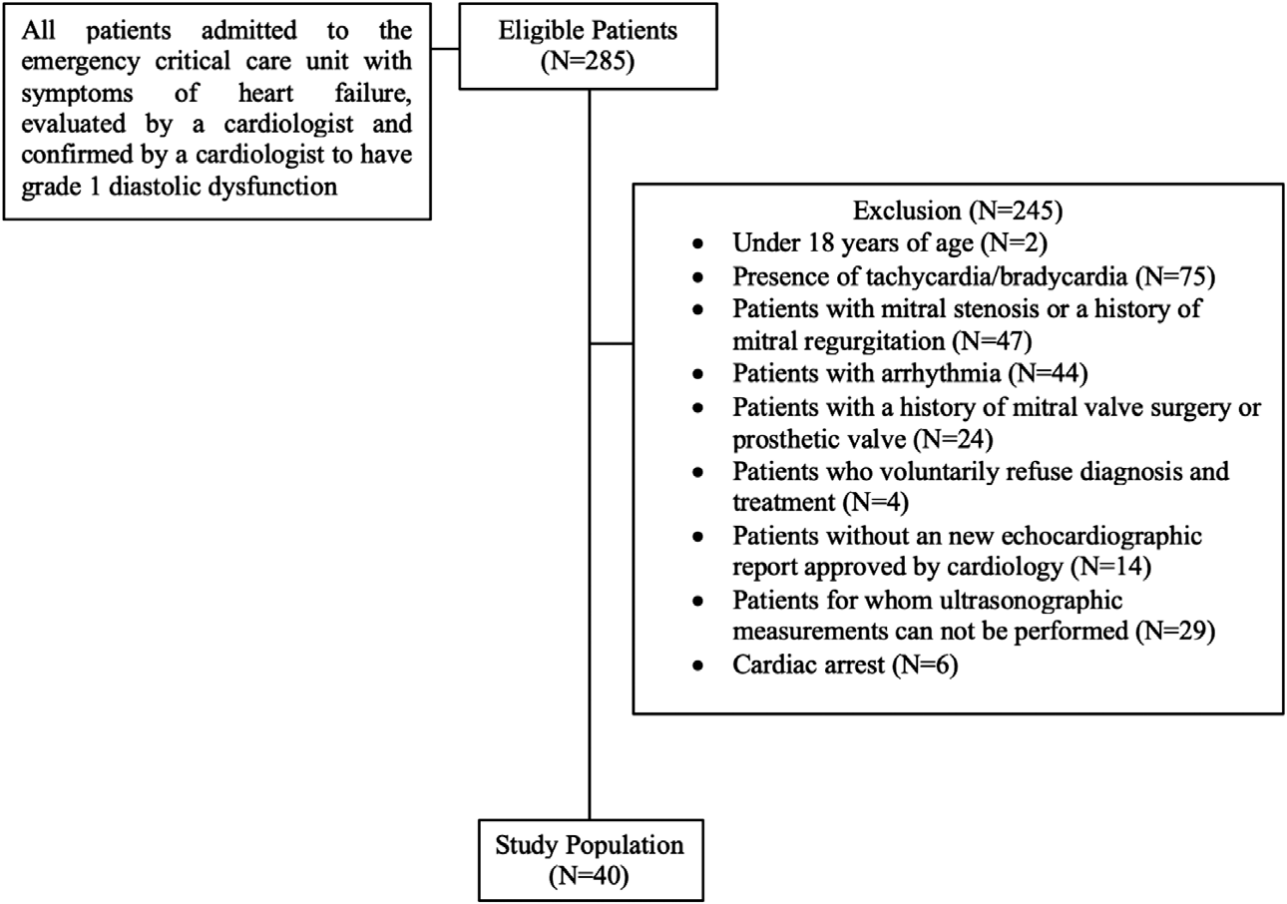
Patient flow chart for enrollment and evaluation of patients with diastolic heart failure with bedside echocardiography in the emergency department.

All patients who presented to the emergency critical care unit with symptoms of heart failure were evaluated by the cardiology department, had grade 1 diastolic dysfunction confirmed by cardiology, and did not meet any of the study’s exclusion criteria. The following patients were excluded: two who were <18 years of age; 75 with tachycardia or bradycardia at presentation; 47 with a history of mitral stenosis or mitral regurgitation; 44 with arrhythmia; 24 with a history of mitral valve surgery; four who refused diagnosis and treatment; 14 who did not have a cardiologist-approved new echocardiography report; 29 for whom ultrasound measurements could not be performed; and six who were brought to the ED because of cardiac arrest. The study population of 40 patients (included rate 14%) was formed after the exclusion criteria were applied to 285 patients who met these conditions. The patient flow chart is shown in Figure 1.

### Study Protocol

Initial patient evaluations in the ED were performed by emergency medicine residents. The study’s patient population was formed primarily based on grade 1 diastolic dysfunction confirmed by a cardiologist, resulting in a group of standardized patients. In the next step, patients with a history of mitral stenosis or mitral regurgitation and a history of mitral valve surgery in echocardiography results who did not have a cardiologist-approved new echocardiography report were excluded. Then, other exclusion criteria were applied. Finally, we excluded patients for whom ultrasonographic measurements were not appropriate and who refused diagnosis and treatment.

The emergency medicine resident performing the initial evaluation recorded all vital and physical examination findings during presentation. The resident also recorded the demographic characteristics, comorbid diseases, and cardiologist-confirmed echocardiography results of the patient he included in the study via the hospital’s automaed system. Primary diagnoses and treatments were made and administered by the emergency medicine resident performing the initial evaluation.

Two emergency physicians who had no responsibility for the patients’ primary care performed the POCUS examinations. Both had participated in and successfully completed ultrasound courses (basic and advanced) that were certified by professional emergency medicine associations (five years of experience in POCUS with an average of 500 ultrasounds per year). They were blinded to the study patients’ laboratory parameters, vital findings, diagnoses, and treatments.

### POCUS Protocol

**Figure 2.**
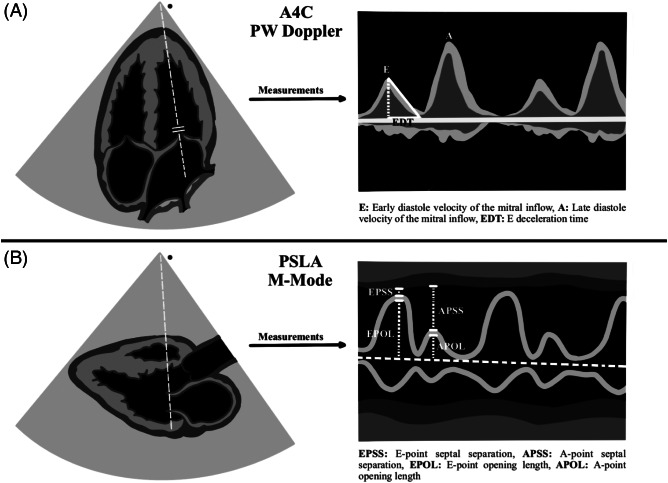
Illustration of measurements. *PSLA*, parasternal long axis; *A4C*, apical four-chamber; *PW*, pulsed wave.

**Figure 3.**
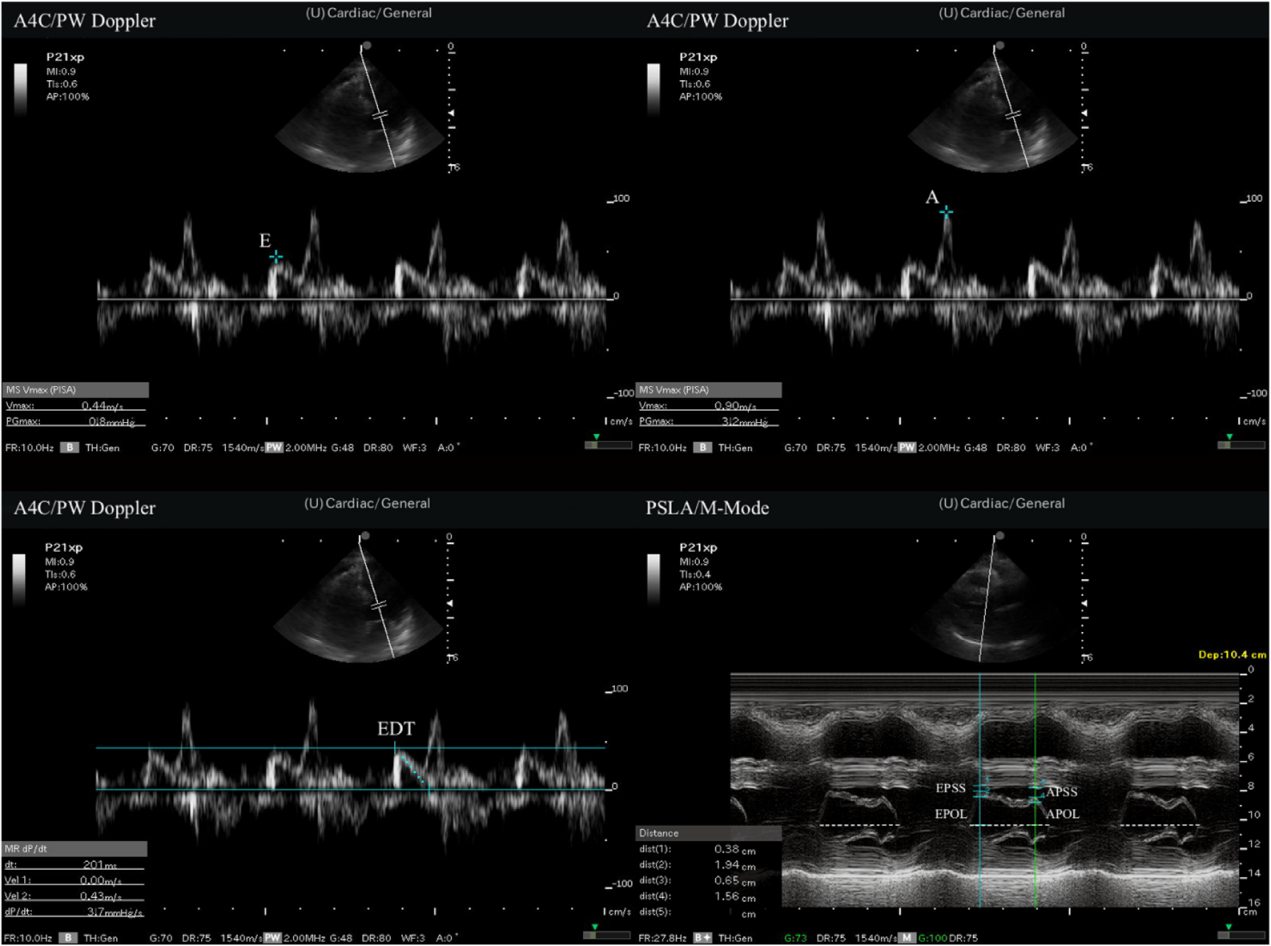
Measurements of point-of-care ultrasound. *A*, late diastole velocity of the mitral inflow; *A4C*, apical 4-chamber; *APOL*, A-point opening length; *APSS*, A-point septal separation; *E*, early diastole velocity of the mitral inflow; *EDT*, E deceleration time; *EPOL*, E-point opening length; *EPSS*, E-point septal separation; *PSLA*, parasternal long axis; *PW*, pulsed wave.

Sonographic examinations were performed on a Fujifilm-Sonosite-FC1 (FUJIFILM SonoSite Inc, Bothell, WA) 2015 model US device. All measurements were made with 1–5 megahertz sector (cardiac) probe. The study protocol included pulsed-wave (PW) Doppler measurements for diastolic function evaluation in A4C view, followed by M-mode measurements via PSLA view scans of the MV anterior leaflet. At sonographic examination, E, A, and E descent time (EDT) measurements were first performed with PW Doppler in A4C view. Then EPSS, A-point septal separation (APSS), A-point opening length (APOL), and E-point opening length (EPOL) were measured with M-mode evaluation at the level of the mitral valve in the PSLA view. The ejection fraction (EF) was calculated using the EPSS method. Measurement time was recorded for both A4C view measurements and PSLA view measurements. The recording procedure commenced once the ultrasonographic windows provided by the images were clearly visible, and it was concluded when the desired measurements had finished. All measurements are summarized in Figures 2 and 3.

### Endpoints

The primary endpoint in this study was to evaluate the correlation between measurements performed with PW Doppler in the A4C view and M-mode measurements in the PSLA view in patients with grade 1 diastolic dysfunction. The secondary outcome point was to compare the time elapsing between the measurements.

### Statistical Analysis

We performed all analyses using Jamovi version 1.6 statistical software (the Jamovi Project 2021, Sydney, Australia). Categorical data were expressed as frequency (n) and percentage. Normally distributed continuous variables were presented as mean plus standard deviation, and non-normally distributed data as median and interquartile range (IQR). Normality of distribution was evaluated using the Shapiro-Wilk test. We compared continuous variables in dependent groups using the paired *t*-test in case of normal distribution and the Wilcoxon test in case of non-normal distribution. The Pearson correlation for normally distributed variables and Spearman correlation analysis for non-normally distributed variables were performed to evaluate the relationships between MV measurements in PSLA view and PW Doppler measurements in A4C view. Since the relationship between MV anterior leaflet M-mode measurements in the PSLA view and Doppler inflow velocity MV measurements had not yet been determined, we did ot perform sample size calculation. *P*-values <0.05 were considered significant for all analyses.

## RESULTS

The study population of 40 patients was constituted following application of the inclusion and exclusion criteria. The study population was formed after all exclusion steps, and ultrasonographic evaluations were performed on all 40 patients. Considering 29 patients evaluated by cardiologists but excluded because of the inability to perform measurements by sonographers, sonographers were able to perform measurements in 40 of 69 patients (58.0%). Twenty-six (65%) of the patients enrolled were men, and 14 (35%) were women. The patients’ mean age was 76.6 years, ranging between 35–97. Hypertension (HT) and coronary artery disease (CAD) were the most common accompanying comorbid diseases.

Analysis of measurements using PW Doppler in the A4C revealed a median E value of 0.5 meters per second (m/s), with an IQR of 0.4–0.6 m/s, median A 0.7 m/s, IQR 0.6–0.8 m/s, and median EDT 258 m/s, IQR 233–279 m/s. Similarly, analysis of measurements taken with M-mode in the PSLA view revealed a median EPSS value of 0.77 centimeters (cm), with an IQR of 0.54–0.89 cm, median APSS 1.10 cm, IQR 0.79–1.31 cm, median EPOL 1.51 cm, IQR 1.23–1.74 cm, and median APOL 1.31 cm, IQR 1.10–1.57 cm. Measurement time was evaluated for both A4C view measurements and PSLA view measurements. The A4C view measurements mean was 70.3 ± 5.4 seconds (sec) to complete, while the PSLA view measurements mean was 44.1 ± 3.8 sec to complete. The patients’ demographic data, initial vital findings, all measurement data, and measurement time are shown in Table 1.

**Table 1.** The patients’ demographic data and baseline characteristics.

Characteristics, N = 40	Value
Gender	
Male, n (%)	26 (65.0%)
Female, n (%)	14 (35.0%)
Age (years), mean ± SD	67.6 ± 13.5
Comorbidities	
Hypertension, n (%)	36 (90.0%)
Diabetes, n (%)	13 (32.5%)
CAD, n (%)	17 (42.5%)
Stroke, n (%)	1 (2.5%)
Dementia, n (%)	3 (7.5%)
Neoplasia, n (%)	7 (17.5%)
CHF, n (%)	1 (2.5%)
Vital signs	
Systolic blood pressure (mmHg), median (IQR)	133 (IQR 130–140)
Diastolic blood pressure (mmHg), median (IQR)	80 (IQR 70–90)
Pulse (/min), median (IQR)	78.5 (IQR 71.8–84)
Measurements	
E (m/s), median (IQR)	0.5 (IQR 0.4–0.6)
A (m/s), median (IQR)	0.7 (IQR 0.6–0.8)
EDT (ms), median (IQR)	258 (IQR 233–279)
EPSS (cm), median (IQR)	0.77 (IQR 0.54–0.89)
APSS (cm), median (IQR)	1.10 (IQR 0.79–1.31)
EPOL (cm), median (IQR)	1.51 (IQR 1.23–1.74)
APOL (cm), median (IQR)	1.31 (IQR 1.10–1.57)
EF (%), median (IQR)	55 (IQR 51.5–60.5)
EPSS/APSS (ratio), median (IQR)	0.70 (IQR 0.64–0.75)
EPOL/APOL (ratio), median (IQR)	1.19 (IQR 1.10–1.29)
E/A (ratio), median (IQR)	0.72 (IQR 0.64–0.78)
Measurement times	
PSLA measurements time (sec), mean ± sd	44.1 ± 3.8
A4C measurements (sec), mean ± sd	70.3 ± 5.4

*A*, late diastole velocity of the mitral inflow; *A4C*, apical 4-chamber; *APOL*, A-point opening length; APSS, A-point septal separation; *IQR*, interquartile range (25p, 75p); *CAD*, coronary artery disease; *CHF*, congestive heart failure; *E*, early diastole velocity of the mitral inflow; *EDT*, E-point deceleration time; *EF*, ejection fraction; *EPSS*, E-point septal separation; *EPOL*, E-point opening length; *PSLA*, parasternal long axis.

**Figure 4.**
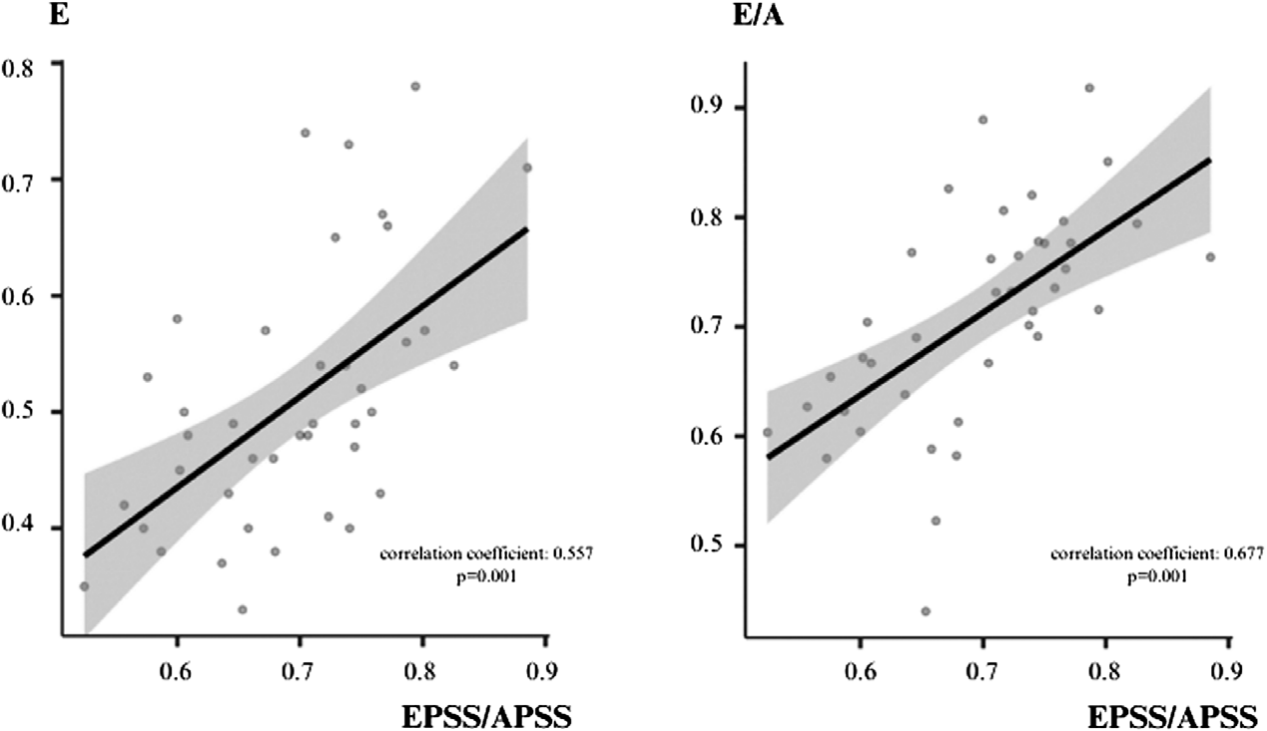
Correlation graphics between measurements in parasternal long axis PSLA view and measurements in apical four-chamber view. *APSS*, A-point septal separation; *E*, early diastole velocity of the mitral inflow; *E/A*, early diastolic velocity of the mitral inflow to the late diastolic velocity ratio; *EPSS*, E-point septal separation.

The correlation between the E/A ratio in PW Doppler measurements and the EPSS/APSS ratio in M-mode was strong (correlation coefficient 0.677, *P* = 0.001). Similarly, the correlation between E in PW Doppler measurements and the EPSS/APSS ratio in M-mode measurements was also moderately strong (correlation coefficient 0.557, *P* = 0.001). No other statistically significant correlations were observed between PW and M-mode measurements. Correlation analysis and graphics between measurements in the PSLA and A4C views is summarized in Table 2 and Figure 4.

**Table 2.** Correlation between measurements in the parasternal long axis view and measurements in the apical four-chamber view.

	Correlation coefficient (*P*-value)			
	E	A	EDT	E/A
EPSS*	−0.036 (0.825)	−0.042 (0.798)	−0.060 (0.711)	0.022 (0.895)
APSS*	−0.277 (0.084)	−0.092 (0.573)	−0.060 (0.712)	−0.236 (0.142)
EPOL*	−0.011 (0.946)	−0.172 (0.288)	−0.034 (0.835)	0.158 (0.331)
APOL*	−0.086 (0.596)	−0.212 (0.189)	0.021 (0.898)	0.074 (0.650)
EPSS/APSS*	0.557 (0.001)	0.090 (0.581)	−0.003 (0.986)	0.677 (0.001)
EPOL/APOL*	−0.079 (0.628)	−0.020 (0.904)	−0.265 (0.098)	−0.033 (0.840)
EF*	0.043 (0.791)	0.081 (0.620)	0.050 (0.759)	−0.053 (0.747)

* Spearman’s correlation analysis.

*APOL*, A-point opening length; *APSS*, A-point septal separation; *E*, early diastole velocity of the mitral inflow; *EDT*, early deceleration time; *EF*, ejection fraction; *EPSS,* E-point septal separation; *EPOL,* E-point opening length; diastole velocity of the mitral inflow.

## DISCUSSION

Echocardiography plays an important role in the evaluation of the systolic and diastolic functions of the left ventricle. It is, therefore, employed in the ED for evaluating both the systolic and diastolic functions of the heart.^12^ ^,^ ^13^ It represents a rapid, repeatable, and non-invasive diagnostic tool for emergency physicians.

Diastolic dysfunction can be seen in conditions such as HT, CAD, and diabetes and can represent a determinant of morbidity and mortality in such diseases.^16^ ^–^ ^18^ The ASE and EACI published updated guidelines to the evaluation of diastolic dysfunction in 2016.^10^ The guidelines recommended the evaluation of four parameters for the diagnosis of diastolic dysfunction: the E/E′ ratio; septal e′ or lateral e′ velocity; tricuspid regurgitation velocity; and the left atrial volume index. It suggested that abnormality should be present in three or four of these parameters for a diagnosis of diastolic dysfunction. These recommendations entail difficulties for the evaluation of patients in the ED setting, one of these being that emergency physicians should be trained in the use of POCUS. The measurements recommended in the guidelines involve complex parameters and measurements for POCUS under ED conditions. More practical diastolic dysfunction evaluation with E, A, and EDT measurement using the PW Doppler method is, therefore, employed in the ED.^11^ ^,^ ^19^ ^,^ ^20^ However, it is also difficult to perform these focused Doppler measurements under ED conditions.

In light of these difficulties, we undertook this study to investigate alternative measurement methods. A different measurement method for evaluating grade 1 diastolic dysfunction was tried by applying M-mode evaluation of MV anterior movement in PSLA view. A statistically strong correlation was thus observed between grade 1 diastolic dysfunction patients’ EPSS/APSS ratios from M-mode measurements in the PSLA view and E/A ratios from PW Doppler measurements in the A4C view (correlation coefficient 0.677, *P* = 0.001). These findings suggest that EPSS and APSS values measured in the ED via M-mode evaluation of MV anterior leaflet movement in the PSLA view may represent a practical approach for diastolic function estimation, similarly to the EPSS method used for systolic function estimation.

Park et al evaluated the relationship between MV anterior leaflet motion in the PSLA view with M-mode measurements and PW Doppler measurements in the A4C view in healthy humans. Those authors determined a significant correlation between the APSS/EPSS ratio and E values (correlation coefficient = 0.4). They concluded that visual evaluation of the M-mode pattern on the MV anterior leaflet in the PSLA view might represent a practical approach toward estimating diastolic function in the ED.^15^ In the present study we evaluated the relationship between M-mode measurements of MV anterior leaflet motion in the PSLA view and PW Doppler measurements in the A4C view in patients with grade 1 diastolic dysfunction. In contrast to the correlation reported by Park et al, we observed positive correlations between the E/A ratio and EPSS/APSS ratio and between E and the EPSS/APSS ratio.

We attribute this discrepancy to the presence of an opposite relationship in the E/A ratio between normal healthy individuals and patients with grade 1 diastolic dysfunction. This contrast may be due to the E/A ratio typically being >1 in healthy individuals, while it falls to <1 with the development of diastolic dysfunction (grade 1). We also think that the positive correlation between the E/A ratio and the EPSS/APSS ratio is associated with physiological compensation developing in grade 1 diastolic dysfunction. This is because in grade 1 diastolic dysfunction, the left ventricular (LV) inflow velocity (E) decreases (passive filling) in early diastole due to impaired LV relaxation, which can lead to an increase in EPSS and a decrease in EPOL. Subsequently, during late diastole, the LV filling rate (A) increases (filling with active propulsion), thus causing an increase in the EPSS/APSS ratio by producing a decrease in APSS. The EPSS/APSS ratio may have exhibited a positive correlation with the E/A ratio as a result of this physiological compensation.

Heart failure remains a major cause of morbidity and mortality. Symptomatic heart failure is due to systolic dysfunction but is also commonly due to diastolic dysfunction.^21^ ^,^ ^22^ The assessment of diastolic dysfunction is of great importance as it is a common cause of symptomatic heart failure. Many emergency physicians do not find the detailed measurements included in the ASE guidelines applicable to POCUS in the ED setting. This is due to multiple factors. For example, if the patient has dyspnea or hypoxemia, they may not be able to lie flat or be properly positioned for the sonographer to obtain an adequate four-chamber view. In addition, many emergency physicians may not have advanced echocardiographic training to perform these measurements. Considering these limitations we thought that a simple method to assess diastolic dysfunction is important for the ED.

We performed this pilot study to demonstrate that a rapid and practical method can be applied in the ED. Our study included only patients with grade 1 diastolic dysfunction, which made it difficult to generalize our study. Considering that grade 1 diastolic dysfunction can be easily affected by the current clinical conception and treatment, among other factors, it suggests that our study should be conducted to include all subgroups of diastolic dysfunction. In addition, because our study included only grade 1 diastolic dysfunction, it included a single-group evaluation. This makes it impossible to calculate the sensitivity or specificity of the M-mode EPSS/APSS ratio measurement. Accordingly, more evidence is needed for the general use of the M-mode EPSS/APSS ratio measurements for grade 1 diastolic dysfunction.

This study also evaluated the time taken for M-mode measurements of the MV anterior leaflet in the PSLA view and PW Doppler measurements in the A4C view. The mean time taken for measurements in the PSLA view was shorter than the mean time taken for measurements in the A4C view. Adopting the time measured after the image window was provided as the starting point eliminated the time difference in finding the appropriate window. The shorter PSLA measurement time suggests that this may be advantageous for diastolic assessment.

## LIMITATIONS

There are a number of limitations to this study. The first is the small study population and the single-center nature of the research. The second is that since the relationship between M-mode measurements of the MV anterior leaflet in the PSLA view and Doppler measurements of the MV inflow velocity in the A4C view is still unclear, sample size calculation could not be performed. It is, therefore, difficult to generalize our results to the wider population. However, we focused on M-mode measurements in the PSLA view and E, A, and EDT measurements in the A4C view, which also permitted a simplified evaluation. Despite the advantages of this approach, it represents another limitation of this study as it does not cover the entire spectrum of diastolic function evaluation. Care was taken to ensure that there was no time between the US evaluation performed by the emergency physicians and the echocardiography evaluation performed by the cardiologists for patient selection, which could have affected the clinical parameters (eg, vital signs) for worsening/improvement. However, the fact that this was not evaluated in terms of time is another limitation of our study. Finally, POCUS was applied by two emergency physicians with experience in ultrasound, and inter-observer agreement was not evaluated. These factors may have affected our results. Further studies with a larger population, including the evaluation of interobserver agreement, are needed to increase the reproducibility and reliability of the diastolic function evaluation method.

## CONCLUSION

When evaluating grade 1 diastolic dysfunction, we found a significant correlation between the M-mode E-point septal separation/A-point septal separation ratio measurement in the parasternal long-axis view and the PW Doppler ratio of the early diastolic velocity of the mitral inflow to the late diastolic velocity measurement in the apical four-chamber window. This association suggests that M-mode measurements in the PSLA may be used in diastolic dysfunction.

## References

[1] Chen L Malek T . Point-of-care ultrasonography in emergency and critical care medicine. Crit Care Nurs Q. 2018;41(2):94–101. 29494365 10.1097/CNQ.0000000000000190

[2] Whitson MR Mayo PH . Ultrasonography in the emergency department. Crit Care. 2016;20(1):227. 27523885 10.1186/s13054-016-1399-x PMC4983783

[3] Gaber HR Mahmoud MI Carnell J et al . Diagnostic accuracy and temporal impact of ultrasound in patients with dyspnea admitted to the emergency department. Clin Ex Emerg Med. 2019;6(3):226–34. 10.15441/ceem.18.072 PMC6774003 31474102

[4] Wright J Jarman R Connolly J et al . Echocardiography in the emergency department. Emerg Med J. 2009;26(2):82–6. 19164613 10.1136/emj.2008.058560

[5] Kuo DC Peacock WF . Diagnosing and managing acute heart failure in the emergency department. Clin Exp Emerg Med. 2015;2(3):141–9. 27752588 10.15441/ceem.15.007 PMC5052845

[6] Massie BM Schiller NB Ratshin RA et al . Mitral-septal separation: new echocardiographic index of left ventricular function. Am J Cardiol. 1977;39(7):1008–16. 868766 10.1016/s0002-9149(77)80215-4

[7] Silverstein JR Laffely NH Rifkin RD . Quantitative estimation of left ventricular ejection fraction from mitral valve E-point to septal separation and comparison to magnetic resonance imaging. Am J Cardiol. 2006;97(1):137–40. 16377299 10.1016/j.amjcard.2005.07.118

[8] Secko MA Lazar JM Salciccioli LA et al . Can junior emergency physicians use E-point septal separation to accurately estimate left ventricular function in acutely dyspneic patients? Acad Emerg Med. 2011;18(11):1223–6. 22044429 10.1111/j.1553-2712.2011.01196.x

[9] Greenstein YY Mayo PH . Evaluation of left ventricular diastolic function by the intensivist. Chest. 2018;153(3):723–32. 29113815 10.1016/j.chest.2017.10.032

[10] Nagueh SF Smiseth OA Appleton CP et al . Recommendations for the evaluation of left ventricular diastolic function by echocardiography: an update from the American Society of Echocardiography and the European Association of Cardiovascular Imaging. J Am Soc Echocardiogr. 2016;29(4):277–314. 27037982 10.1016/j.echo.2016.01.011

[11] Oh JK Park SJ Nagueh SF . Established and novel clinical applications of diastolic function assessment by echocardiography. Circ Cardiovasc Imaging. 2011;4(4):444–55. 21772012 10.1161/CIRCIMAGING.110.961623

[12] Unlüer EE Bayata S Postaci N et al . Limited bedside echocardiography by emergency physicians for diagnosis of diastolic heart failure. Emerg Med J. 2012;29(4):280–3. 21441267 10.1136/emj.2011.111229

[13] Ehrman RR Russell FM Ansari AH et al . Can emergency physicians diagnose and correctly classify diastolic dysfunction using bedside echocardiography? Am J Emerg Med. 2015;33(9):1178–83. 26058890 10.1016/j.ajem.2015.05.013

[14] Konecke LL Feigenbaum H Chang S et al . Abnormal mitral valve motion in patients with elevated left ventricular diastolic pressures. Circulation. 1973;47(5):989–96. 4705588 10.1161/01.cir.47.5.989

[15] Park CH Yoon H Jo IJ et al . A pilot study evaluating LV diastolic function with M-mode measurement of mitral valve movement in the parasternal long axis view. Diagnostics (Basel). 2023;13(14):2412. 37510155 10.3390/diagnostics13142412 PMC10378499

[16] Vasan RS Benjamin EJ Levy D . Prevalence, clinical features and prognosis of diastolic heart failure: an epidemiologic perspective. J Am Coll Cardiol. 1995;26(7):1565–74. 7594087 10.1016/0735-1097(95)00381-9

[17] Fiack CA Farber HW . Heart failure with preserved ejection fraction. N Engl J Med. 2006;355(17):1828–31. 10.1056/NEJMc062229 17065647

[18] Owan TE Hodge DO Herges RM et al . Trends in prevalence and outcome of heart failure with preserved ejection fraction. N Engl J Med. 2006;355(3):251–9. 16855265 10.1056/NEJMoa052256

[19] King SA Salerno A Downing JV et al . POCUS for diastolic dysfunction: a review of the literature. POCUS J. 2023;8(1):88–92. 37152335 10.24908/pocus.v8i1.15803 PMC10155731

[20] Li Y Yin W Qin Y et al . Preliminary exploration of epidemiologic and hemodynamic characteristics of restrictive filling diastolic dysfunction based on echocardiography in critically ill patients: a retrospective study. Biomed Res Int. 2018;2018:5429868. 29682549 10.1155/2018/5429868 PMC5841041

[21] Abbate A Arena R Abouzaki N et al . Heart failure with preserved ejection fraction: refocusing on diastole. Int J Cardiol. 2015;179:430–40. 25465302 10.1016/j.ijcard.2014.11.106

[22] Yancy CW Lopatin M Stevenson LW et al . Clinical presentation, management, and in-hospital outcomes of patients admitted with acute decompensated heart failure with preserved systolic function: a report from the Acute Decompensated Heart Failure National Registry (ADHERE) database. J Am Coll Cardiol. 2006;47(1):76–84. 16386668 10.1016/j.jacc.2005.09.022

